# A Atividade Física no Presente Pode Ser a Receita para Evitar os Males da Obesidade e Hipertensão no Futuro

**DOI:** 10.36660/abc.20200483

**Published:** 2020-07-28

**Authors:** Roberto Jorge da Silva Franco

**Affiliations:** Universidade Estadual Paulista Júlio de Mesquita Filho Faculdade de Medicina BotucatuSP Brasil Universidade Estadual Paulista Júlio de Mesquita Filho Câmpus de Botucatu Faculdade de Medicina, Botucatu, SP – Brasil

**Keywords:** Hipertensão, Fatores de Risco, Doenças Cardiovasculares/mortalidade, Adolescentes, Adulto, Obesidade, Atividade Física, Sedentarismo

*Minieditorial referente ao artigo: Medidas Hipertensivas em Escolares: Risco da Obesidade Central e Efeito Protetor da Atividade Física Moderada-Vigorosa*

A hipertensão arterial é uma das causas mais importantes de morte prematura no mundo. Quase um bilhão de pessoas são afetadas e estima-se que até 2025, 1,5 bilhão de adultos terão HA.^1^ Felizmente, a HA é um dos principais fatores de risco modificáveis para doenças cardiovasculares com raízes na infância, tornando evitáveis tanto o desenvolvimento da doença quanto o óbito.

Atualmente, a prevalência de HA em crianças e adolescentes tornou-se um importante problema de saúde pública.^2^ Um exemplo de como esse cenário se agravou é a comparação de um estudo feito pelo nosso grupo^3^ com duas coortes sucessivas (1975–1976) de estudantes de 16 a 25 anos de idade da cidade de Botucatu, estado de São Paulo, em que encontraram uma prevalência de HA de 5,0% e 6,2%, respectivamente, além de outro estudo mais recente. Com quase 40 anos de atraso, o estudo brasileiro sobre riscos cardiovasculares em adolescentes (ERICA),^4^ um estudo escolar nacional que contou com 73.999 adolescentes com idades entre 12 e 17 anos, encontrou uma prevalência de HA de 9,6%. Os adolescentes obesos apresentaram maior prevalência de hipertensão — 28,4% — que os adolescentes com excesso de peso, com 15,4%, e os eutróficos, com 6,3%. A fração de HA atribuível à obesidade foi de 17,8%.

Muitas razões podem explicar a crescente prevalência de HA em jovens. A obesidade é a principal, seguida pelo consumo de sal e açúcar, ambientes estressantes, baixo nível de atividade física e estilo de vida sedentário. No Brasil, um estudo transversal sobre consumo de alimentos^5^ em amostra representativa da população com 10 anos ou mais de idade observou que o maior consumo de alimentos ultraprocessados estava associado a maior teor de gorduras em geral, gordura saturada, gordura trans e açúcares livres.

As evidências da proteção contra o desenvolvimento de HA e doenças cardiovasculares e mortalidade por todas as causas por parte da atividade física regular tornaram-se incontestáveis.^6^ A maioria das pessoas em todas as sociedades industrializadas está se tornando menos ativa fisicamente em suas vidas diárias, passando cada vez mais tempo fazendo atividades sedentárias. O aumento da atividade física e níveis mais elevados de capacidade de exercício não só reduzirão o risco de desfechos cardiovasculares e diabetes, como também impedirão o desenvolvimento de hipertensão.^7^ A incidência de HA mostrou-se reduzida em 28% entre homens e 35% entre mulheres em atividade física, como corrida ou natação, em um seguimento prospectivo de 11 anos, com mais de 12.000 finlandeses.^8^ Em estudo prospectivo longitudinal,^9^ avaliamos a associação entre o nível de atividade física e mortalidade em 200 pacientes hipertensos e diabéticos em 2012 e reavaliados em 2018. Durante 6 anos de seguimento, 80% dos pacientes ativos sobreviveram em comparação aos sedentários. Além disso, observou-se o benefício da atividade física em pessoas irregularmente ativas, com 65% de chances de sobreviver em comparação com pacientes que não mantêm essa prática saudável.

O artigo “Medidas Hipertensivas em Escolares: Risco da Obesidade Central e Efeito Protetor da Atividade Física Moderada-Vigorosa”,^10^ publicado nesta revista, tem como objetivo verificar a associação de HA, obesidade central e geral e o nível de atividade física em crianças em idade escolar e adolescentes. O estudo envolveu 336 crianças e adolescentes, com idades entre 11 e 17 anos. Foram medidos a altura, peso, circunferência abdominal (CA) e pressão arterial (PA). Calculou-se o escore Z do índice de massa corporal (IMC-z). O nível de atividade física foi avaliado pela versão curta do Questionário Internacional de Atividade Física ( *International Physical Activity Questionnaire* — IPAQ), de acordo com a prática de atividades físicas moderadas a vigorosas (AF-mv). Crianças com PA sistólica (PAS) ou PA diastólica (PAD) acima do percentil 95, de acordo com sexo, idade e altura ou acima de 120/80 mmHg, foram consideradas hipertensas. A análise estatística foi avaliada pelo teste t de Student, qui-quadrado, Mann-Whitney e modelo de regressão logística binária, onde p<0,05 foi o nível de significância. Encontrou-se HA em 40,5% dessa população, sobrepeso em 31,1% e obesidade em 12,5%, sendo 13,4% com maior CA. Crianças insuficientemente ativas em AF-mv representavam 40,2%. A HA esteve associada a maior CC (OR=6,1; IC 95%: 2,6 a 14,4) e sobrepeso (OR=2,9; CI 95%: 1,8 a 4,5). Além disso, as crianças com prática de AF-mv apresentaram menor risco de PAD elevada (OR=0,33; IC 95%: 0,15 a 0,72), redução de risco em 33%. Em conclusão, obesidade central, obesidade em geral e sexo masculino foram melhores preditores de HA. A prática de AF-mv protege as crianças em idade escolar contra o desenvolvimento de hipertensão diastólica.

A obesidade infantil e adolescente é seguida de obesidade na idade adulta e tem estado implicada em diversas doenças crônicas, como diabetes tipo 2, hipertensão e doenças cardiovasculares, e ligada à mortalidade e óbito prematuro. Por esses motivos, nós compartilhamos das mesmas percepções expressas por Tozo et al.,^10^ que deixam clara a relevância da obesidade e da hipertensão na infância e na adolescência. Embora a prevalência da hipertensão não seja o objetivo do estudo, é incomum em comparação com o estudo ERICA.^5^ No entanto, o mérito do estudo é a informação preciosa que alerta e nos chama a abrir os olhos para o futuro próximo, enfatizando a importância de fatores crescentes de doenças cardiovasculares como obesidade, hipertensão, estilo de vida sedentário, maus hábitos alimentares e inatividade física que assolam a nossa juventude. Recentemente, aprendemos que os mesmos fatores de risco para doenças cardiovasculares também são os mesmos para doenças causadas por infecção por vírus.^11^ Está na hora de nos mexermos. A mensagem final é: Olhe para o futuro para tomar as decisões corretas no momento, para não se arrepender das consequências causadas pela obesidade e hipertensão na juventude.
