# Supplementary material for: A Atividade Física no Presente Pode Ser a Receita para Evitar os Males da Obesidade e Hipertensão no Futuro
Source: Arq Bras Cardiol. 2020 Jul 28;115(1):50–1. [Article in Portuguese] doi: 10.36660/abc.20200483 (PMC8384312; doi:10.36660/abc.20200483)
Supplement: Translation

# Translation: Physical Activity in the Present Can Be the Recipe to Avoid the Ills of Obesity and Hypertension in the Future

**Authors:** Roberto Jorge da Silva Franco

**Affiliations:** Brazil Universidade Estadual Paulista Júlio de Mesquita Filho Campus de Botucatu Faculdade de Medicina, Botucatu, SP – Brazil

**Keywords:** Hypertension, Risk Factors, Cardiovascular Diseases/mortality, Adolecents, Adult, Obesity, Physical,Activity, Sedentarism

*Short Editorial related to the article: Hypertensive Measures In Schoolchildren: Risk Of Central Obesity And Protective Effect Of Moderate-To-Vigorous Physical Activity*

Arterial hypertension is one of the most important causes of premature death in the world. Nearly one billion are affected and it is estimated that by 2025, 1.5 billion adults will be living with AH.^1^ Fortunately, AH is a major modifiable risk factor for cardiovascular diseases with roots in childhood making both the development of the disease and death to be preventable.

Nowadays, the prevalence of AH in children and adolescents has become a significant public health issue.^2^ One example of how this scenario has become worse is the comparison of a study conducted by our group,^3^ with two successive cohorts (1975–1976) of students aged 16 to 25 from the city of Botucatu, São Paulo state, which found the prevalence of AH of 5.0% and 6,2%, respectively, with another more recent study. Almost 40 years late, the Brazilian study of cardiovascular risks in adolescents (ERICA),^4^ a national school-based study involving 73,999 adolescents aged 12 to 17, found an AH prevalence of 9.6%. Obese adolescents presented higher prevalence of hypertension — 28.4% — than overweight adolescents, with 15.4%, or eutrophic adolescents, with 6.3%. The portion of AH attributable to obesity was 17.8%.

Many reasons may explain the growing prevalence of AH in young people. Obesity is the main one, followed by consumption of salt and sugar, stressful environments, low level of physical activity and sedentary lifestyle. In Brazil, a cross-sectional study on food consumption^5^ in a representative sample of a population aged 10 or older noted that higher consumption of ultraprocessed food was associated with higher content of fats in general, saturated fat, trans fat and free sugars.

Evidence of protection against both the development of AH and cardiovascular diseases and all-cause mortality by regular physical activity has become incontestable.^6^ Most people in all industrialized societies are becoming less physically active in their daily lives, spending more and more time in sedentary activities. Not only will increased physical activity and higher levels of exercise capacity reduce the risk of cardiovascular outcomes and diabetes, but they will also prevent the development of hypertension.^7^ The incidence of AH was reduced by 28% in men and 35% in women doing physical activities such as jogging or swimming in a prospective 11-year follow-up of over 12,000 Finish people.^8^ In a prospective longitudinal study,^9^ we evaluated the association between level of physical activity and mortality in 200 hypertensive and diabetic patients in 2012 and reassessed it in 2018. Over 6 years of follow-up, 80% of active patients survived compared to sedentary patients. Also, the benefit of physical activity was seen in irregularly active people, with 65% of chances to survive compared to patients who do not maintain this healthy practice.

The article “ *Hypertensive Measures In Schoolchildren: Risk Of Central Obesity And Protective Effect Of Moderate-To-Vigorous Physical Activity* ”^10^ now reported in this journal has the aim of verifying the association of AH, central and general obesity and level of physical activity in schoolchildren and adolescents. The trial involved 336 children and adolescents aged 11–17. Height, weight, waist circumference (WC) and blood pressure (BP) were measured. Body mass index score Z (BMI-z) was calculated. The level of physical activity was evaluated by the short International Physical Activity Questionnaire (IPAQ), according to practice of moderate-vigorous physical activities (PA-mv). Schoolchildren with systolic blood pressure (SBP) or diastolic blood pressure (DBP) above the 95^th^percentile, according to sex, age and height, or over 120/80 mmHg, were considered hypertensives. Statistical analysis was evaluated by the Student’s t test, Chi-square, Mann-Whitney and binary logistic regression model, where p<0.05 was the significant level. AH was found in 40.5% of this population, of which 31.1% were overweight and 12.5% were obese, and 13,4% had large WC. Schoolchildren insufficiently active in PA-mv accounted for 40.2%. AH was associated to higher WC (OR=6.1; 95% CI: 2.6 to 14.4) and to overweight (OR=2.9 95% CI: 1.8 to 4.5). Besides that, schoolchildren practicing PA-mv presented lower risk of high DBP (OR=0.33; 95% CI: 0.15 to 0.72) and risk reduction of 33%. In conclusion, central obesity, general obesity and male sex were better predictors of AH. Practicing PA-mv protects schoolchildren from developing diastolic hypertension.

Obesity in childhood and adolescence is followed by obesity in adult age and has been implicated in many chronic diseases, including type 2 diabetes, hypertension and cardiovascular diseases, and linked to mortality and premature death. For these reasons, we empathize with the paper by Tozo et al.,^10^ who make clear the relevance of obesity and hypertension in childhood and adolescence. Although the prevalence of hypertension is not the focus of the study, it is unusual compared to the ERICA study.5 However, the merit of the study is that it provides precious information, warning us and calling us to open our eyes to the near future stressing the importance of growing factors of cardiovascular diseases such as obesity, hypertension, sedentary lifestyle, bad eating habits and physical inactivity that adversely affect our youth. We have recently learned that the same risk factors for cardiovascular diseases are also the same for virus infection deseases.^11^ It is time to move. The final message is: look to the future to make the right decisions at the right moment so as not to regret the consequences caused by obesity and hypertension in youth.
